# Clinical learning in the context of uncertainty: a multi-center survey of emergency department residents’ and attending physicians’ perceptions of clinical feedback

**DOI:** 10.1186/s12909-019-1597-8

**Published:** 2019-05-29

**Authors:** Chung-Hsien Chaou, Yu-Che Chang, Shiuan-Ruey Yu, Hsu-Min Tseng, Cheng-Ting Hsiao, Kuan-Han Wu, Lynn Valerie Monrouxe, Roy Ngerng Yi Ling

**Affiliations:** 1 Chang-Gung Medical Education Research Centre, Chang Gung Memorial Hospital, Taoyuan, Taiwan; 2 Department of Emergency Medicine, Chang Gung Memorial Hospital, Linkou and Chang Gung University College of Medicine, Taoyuan, Taiwan; 3 Department of Health Care Management, Chang Gung University, Taoyuan, Taiwan; 4 Department of Emergency Medicine, Chang Gung Memorial Hospital, Chiayi and Chang Gung University College of Medicine, Taoyuan, Taiwan; 5 Department of Emergency Medicine, Chang Gung Memorial Hospital, Kaohsiung and Chang Gung University College of Medicine, Taoyuan, Taiwan

**Keywords:** Feedback, Residency, Postgraduate training, Questionnaire survey, Emergency department

## Abstract

**Background:**

Feedback is an essential part of clinical teaching and learning, yet it is often perceived as unsatisfactory in busy clinical settings. Clinical teachers need to balance the competing demands of clinical duty and feedback provision. The influence of the clinical environment and the mutual relationship between feedback giving and seeking has been inadequately investigated. This study therefore aimed to quantify the adequacy, perceptions, and influential factors of feedback provision during resident training in emergency departments (EDs).

**Methods:**

A multicenter online questionnaire study was undertaken. The respondents comprised ED residents and clinical teachers from four teaching hospitals in Taiwan. The questionnaire was developed via an expert panel, and a pilot study ensured validity. Ninety clinical teachers and 54 residents participated.

**Results:**

The respondents reported that the majority of feedback, which usually lasted 1–5 min, was initiated by the clinical teachers. Feedback satisfaction was significantly lower for the clinical teachers than for the residents (clinical teachers M = 13.8, SD = 1.83; residents M = 15.3, SD = 2.14; *p* < 0.0001), and positive feedback was provided infrequently in clinical settings (31.1%). Both groups of participants admitted hesitating between providing/seeking feedback and completing clinical work. Being busy, the teachers’ clinical abilities, the learners’ attitudes, and the relationship between both parties were reported as the most influential factors in feedback provision.

**Conclusion:**

ED clinical feedback provision is often short, circumstantial, and initiated by clinical teachers. Providing or seeking feedback appears to be an important part of clinical learning in the context of uncertainty. The importance of the relationship between the feedback seeker and the provider highlights the interactive, reciprocal nature of clinical feedback provision.

**Electronic supplementary material:**

The online version of this article (10.1186/s12909-019-1597-8) contains supplementary material, which is available to authorized users.

## Background

Feedback is an essential part of modern clinical teaching and learning [1, 2] and is a good pedagogical tool for the development of different levels of learners and across different healthcare professional settings [3, 4]. Receiving appropriate and timely feedback helps clinical learners identify their strengths and weaknesses within different competency domains [5]. Both positive and negative verbal feedback can be potent stimulants for the improvement of performance and motivation [6]. However, although most clinicians are familiar with the importance of giving feedback, many do not recognize the plethora of opportunities presented to them for which they could use feedback as a teaching tool [7].

Residency is a pivotal training period during which a learner’s self-identity gradually shifts from one of a medical student to that of a specialized doctor [8]. Learning during this period is very different from the undergraduate setting and is characterized by competency-based, case-oriented, and hands-on authentic learning [9]. Several important non-knowledge-based clinical competencies, such as system-based practice and leadership skills, are also learned during this period [10]. Research suggests that high-quality work-based feedback improves resident performance [7, 11]. Nevertheless, feedback for residents in a busy clinical setting is often challenging and perceived as unsatisfactory [12].

The emergency department (ED) is a rich learning environment in which patients present with all kinds of symptoms and diseases. It also introduces residents to a range of clinical procedures, inter-professional and interpersonal communications, and leadership styles [10]. Supervision in the ED is characterized by its availability. Teachers and junior doctors work together, or in parallel, forming a unique environment to facilitate feedback provision and seeking. Previous studies have shown that feedback in the ED stimulates learning motivation [6], helps the generation and execution of learning goals [13], and improves medical knowledge and skills [14]. On the other hand, feedback provision in the ED is often hindered by its fast-paced nature, unpredictable educational conditions, and frequent interruptions [15–18]. In a recent qualitative study addressing the challenges of clinical feedback within the ED, clinical teachers revealed how they were continually striving to keep a balance between providing feedback to learners and maintaining patient safety [19]. However, the prevalence of such behaviors and other contributing factors that facilitate and inhibit feedback provision in a busy clinical environment has not yet been adequately investigated and quantified.

Feedback seeking has also been recognized as a good educational method amongst learners [20–23]. Recent studies have shown that residents regard actively engaging in feedback-seeking as an effective way of learning within the workplace [21, 24]. Indeed, medical students recognize the complex relationship between their own behavior and that of their trainers [22]. As such, they use and seek feedback during their professional learning through what some researchers have conceptualized as an “educational alliance” [23, 25]. To date the study of clinical feedback among learners and teachers has rarely focused on the influences of clinical burden and mutual relationships. The current study aimed to address this gap by quantifying clinical teachers and residents’ viewpoints on the adequacy, current perceptions, and clinical influential factors on feedback provision during resident training in a busy clinical setting.

## Methods

### Study design

A multicenter questionnaire survey was undertaken. The respondents comprised emergency department (ED) residents and ED clinical teachers. The questionnaire was live online between March and June 2016 and was approved by the hospital institutional review board (IRB No. 104-9479B). Written consent was obtained from all the participants.

### Study setting and population

The survey was conducted in the EDs of four branches of a large teaching hospital group in Taiwan. The selected hospitals differed in their geographical locations, hospital accreditation levels, number of daily ED patient visits, and faculty capacities. A summary of the four study sites is presented in Table 1. The total number of clinical teachers and residents across these branches was 119 and 62, respectively. In the present Taiwanese medical education system, a learner enters medical school after high school graduation for a seven-year undergraduate curriculum. This is followed by a year of postgraduate rotational training, four years of emergency medicine resident training, and one year of optional fellowship before becoming an ED attending physician (the equivalent of a consultant in some countries).

**Table 1:** Study site demographics

	Accreditation level	Annual ED census	Number of Clinical teachers	Number of residents
Site A	Regional	76,000	18	8
Site B	Medical center	181,000	54	28
Site C	Regional	77,000	18	9
Site D	Medical center	141,000	29	17

### Questionnaire development and data collection

A thorough literature review on clinical feedback was conducted prior to the questionnaire development. We developed the questionnaire items based on existing articles focusing on feedback provision within the ED. [12, 15, 19, 26] Further, Yarris et al. [15] reported a gap between the perceptions of the clinical teachers and residents in their study regarding feedback quality, timelines, and frequency, thus two versions of our questionnaire were designed (one for each group of participants) for better comparison. An expert panel of ED physicians and questionnaire development specialists (i.e., CHC, SRY, SMT, YCC, and two non-author experts listed in the acknowledgements) was formed for the questionnaire development phase. The results of the literature review were provided to this panel. Two meetings were held to formulate the questionnaire items regarding perception measurement and possible influential factors. To avoid ambiguity, a four-point Likert scale was selected to measure the degree of perceptions and the effects of barriers and facilitators. Following the initial questionnaire development, a pilot study comprising six residents and six clinical teachers was undertaken. The pilot study took the form of one-to-one face-to-face sessions with an observer looking for any difficulties occurring during the questionnaire completion process. The observer also noted the time required for the completion of the questionnaire.

Two forms of the questionnaire, one paper-based and one web-based, with the same content were initially formulated. The web-based format was undertaken using typeform.com. They were both provided, in random order, during the pilot study, and feedback was sought by the observer. The web-based format was eventually chosen because of its more user-friendly interface, hand-held device accessibility, and pop-up alert to minimize the potential for respondents to miss values. To avoid confusion, and as suggested by the expert panel, a definition and examples of clinical feedback were provided to the participants at the beginning of the questionnaire. Although other definitions have been proposed in the literature [1, 18, 27, 28]. the operational definition of clinical feedback provided by van de Ridder et al. [29] was used due to its wide acceptability:

“Specific information about the comparison between a trainee's observed performance and a standard, given with the intent to improve the trainee's performance.”

An English translation of the two versions of the questionnaire is provided in Additional file 1. All the clinical teachers and residents from the four EDs were invited to participate via e-mail.

### Data analysis

For the descriptive results, the mean (M) and standard deviation (SD) were used to describe the central tendency and the spread of the survey results. The internal consistency was checked using Cronbach’s alpha. A result of > 0.8 indicated a good internal consistency. The mean sum of the satisfactory scores among the two groups of participants were compared using an independent *t*-test, and the individual item scores were compared using the Wilcoxon rank sum test [30]. The categorical or ordinal results between the groups were compared using the chi-square test or Fisher’s exact test, as appropriate. The analyses were performed using SAS statistical software version 9.3 [31].

## Results

### Descriptive results of the participants

Ninety clinical teachers (75.6% response rate) and 54 residents (87.1% response rate) participated in the survey. The average ages of the clinical teachers and residents were 40.0 years and 29.9 years, respectively. About half (45%) of the clinical teachers had previously attended a feedback training course, and 17% held a university teaching affiliation. A detailed list of the number of participants for each branch and their seniority is presented in Table 2. The Cronbach’s alpha coefficient for the clinical teachers and the residents’ versions of the questionnaire were 0.87 and 0.85, respectively, indicating a good internal consistency.

**Table 2:** Descriptive results of the participants

			Clinical teachers (*n* = 90)		Residents (*n* = 54)		*p*-value
Male gender			75	(83.3)	48	(88.9)	0.3605
Age			40.2	(5.47)	29.9	(1.51)	< 0.0001
Branch							0.8279
Site A			45	(50.0)	25	(46.3)	
Site B			14	(15.6)	7	(13.0)	
Site C			12	(13.3)	7	(13.0)	
Site D			19	(21.1)	15	(27.8)	
Seniority (Attending / Residents)							NA
≦5 yrs.	/	1st year	28	(31.1)	12	(22.2)	
6 -10 yrs	/	2nd year	27	(30.0)	13	(24.1)	
11-15 yrs	/	3rd year	24	(26.7)	17	(31.5)	
16-20 yrs	/	4th year	8	(8.89)	12	(22.2)	
> 20 yrs	/	5th year	3	(3.33)	0	(0)	
Initiation of the feedback^a^							< 0.0001
Mostly by Clinical teacher			50	(55.6)	6	(11.1)	
Mostly by Residents			3	(3.33)	4	(7.40)	
About fifty-fifty			37	(41.1)	44	(81.5)	
Feedback duration (each)^a^							0.0003
Less than 1 min			3	(3.33)	4	(7.40)	
1 to 5 min			73	(81.1)	42	(77.8)	
5 to 10 min			2	(2.22)	8	(14.8)	
More than 10 min			12	(13.3)	0	(0)	
Feedback frequency							0.2623
≥ 5 per shift			32	(35.6)	13	(24.1)	
< 5 per shift			50	(55.6)	33	(61.1)	
Once every 2–3 shifts			8	(8.89)	7	(13.0)	
Once every > 3 shifts			0	(0)	1	(1.85)	
Attended feedback training course			45	(50.0)			NA
University affiliated			15	16.7			NA

^a^Statistically significant difference detected between groups using Chi-square test, Fisher exact test, or independent T test, when appropriate

### Perceptions of clinical feedback provision among the participants

Over half (56%) of the clinical teachers reported that they initiated the feedback process, but most residents (82%) reported that there was a 50/50 chance of initiation from both sides. The duration of each feedback was primarily between 1 and 5 min, with over 80% of both participant groups agreeing that feedback occurred at least once in every clinical shift. Both groups of participants reported the occurrence of positive feedback as the least frequent event (31% for the clinical teachers, 9% for the residents).

**Fig. 1:**
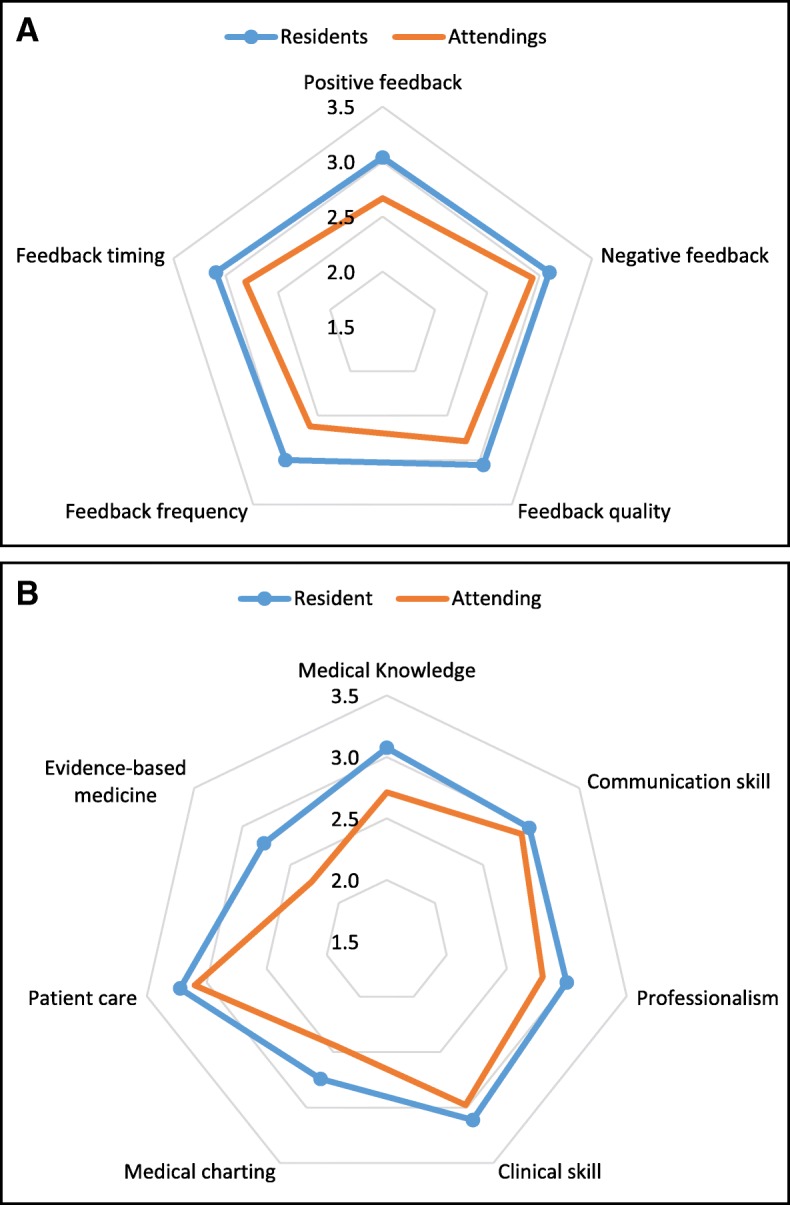
Perceptions of clinical feedback provision among the residents and clinical teachers in terms of (**a**) overall satisfaction and (**b**) specific feedback content

Figure 1 displays the differing perceptions of feedback provision among the participant groups. Their overall satisfaction across five domains is shown in Fig. 1a, while Fig. 1b displays the responses to seven items with a specific focus on the content of the clinical feedback. The mean sum scores of overall satisfaction in the clinical teachers’ group were significantly lower than those in the residents’ group (clinical teachers M = 13.8, SD = 1.83; residents M = 15.3, SD = 2.14; *p* < 0.0001). Similar findings of the mean sum scores were also identified for the content-specific feedback domains (clinical teachers M = 19.2, SD = 2.80; residents M = 20.9, SD = 2.20; *p* < 0.01). Furthermore, a larger difference was found between the two groups for the content-specific feedback item related to evidence-based medicine (clinical teachers M = 2.28, SD = 0.67; residents M = 2.78, SD = 0.66; *p* < 0.0001).

### Feedback triggers

Table 3 displays the results associated with the different triggers for which feedback was provided. The participants were asked to indicate the largest range of situations in which they would provide/receive feedback. The most common situation reported by both groups was when a resident takes the initiative to ask for feedback (94.4% for the clinical teachers, 89.9% for the residents, *p* = 0.2243). The situations that showed the most significant difference between the clinical teachers and residents were “When the resident’s decision can cause harm to the patient” (91.1% vs 66.7%, respectively, *p* < 0.001), “When there are communication problems” (75.6% vs 35.2%, respectively, *p* < 0.0001), and “After the resident’s initial evaluation of the patient” (63.3% vs 31.5%, respectively, *p* < 0.001).

**Table 3:** Triggers for feedback provision in the clinical ED setting

	Clinical teacher (*n* = 90)		Residents (*n* = 54)		*p*-value
	N	(%)	N	%	
When the resident takes the initiative to seek feedback	85	(94.4)	48	(88.9)	0.2243
When the resident’s decision can cause harm to the patient^a^	82	(91.1)	36	(66.7)	0.0002
When there are changes to the patient’s condition	71	(78.9)	39	(72.2)	0.3618
When deciding on the patient’s disposition	71	(78.9)	48	(88.9)	0.1251
When there are new findings to the patient’s examination results	69	(76.7)	34	(63.0)	0.0777
When there are communication problems^a^	68	(75.6)	19	(35.2)	< 0.0001
When encountering unusual or rare medical cases	68	(75.6)	40	(74.1)	0.8424
After medical orders were given by the resident^a^	67	(74.4)	28	(51.9)	0.0056
After the resident’s initial evaluation of the patient^a^	57	(63.3)	17	(31.5)	0.0002
After a resident was observed to perform well^a^	28	(31.1)	5	(9.26)	0.0025

^a^Significantly different between two groups of participants. Comparison was done using Chi-square test

### Influential factors in clinical feedback provision

When responding to the items focusing on the influential factors related to clinical feedback provision, 57% of the clinical teachers and 61% of the residents (*p* = 0.600) reported that they struggled with the tension between providing/seeking feedback and finishing their clinical tasks. Further, 76% of the clinical teachers reported that they actually *forgot* to give the residents feedback when concentrating on their work. Around half of the respondents (clinical teachers 52%, residents 52%, *p* = 0.966) reported a willingness to leave work late so that they could provide or receive feedback. Only 30% of the clinical teachers reported feeling troubled with having to adapt to different learners on a daily basis. On the other hand, up to 50% of the residents reported having difficulty in adapting to different teachers.

**Fig. 2:**
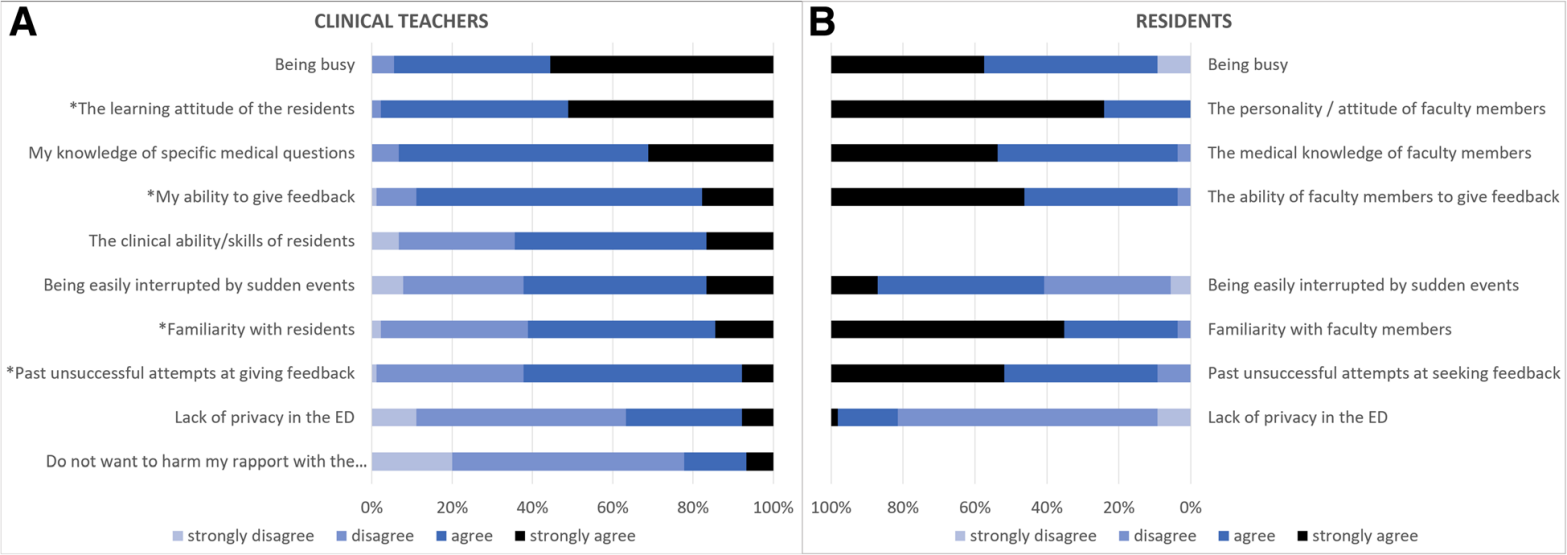
Perceptions of the influential factors affecting the willingness to provide or seek feedback: (**a**) responses from the clinical teachers (faculty members), (**b**) responses from the residents. * Statistically significant difference using Wilcoxon rank-sum test

Figure 2 presents a comparison of the clinical teachers and residents’ perceptions of the various influential factors impacting their willingness to provide and seek feedback.

Statistically significant differences were found in the areas of personal attitude (clinical teacher M = 3.49, SD = 0.55; residents M = 3.76, SD = 0.43; *p* < 0.01), teachers’ ability to provide feedback (clinical teachers M = 3.06, SD = 0.57; residents M = 3.50, SD = 0.57; *p* < 0.0001), familiarity with each other (clinical teachers M = 2.73, SD = 0.73; residents M = 3.61, SD = 0.56; *p* < 0.0001), and past unsuccessful attempts to deliver/request feedback (clinical teachers M = 2.69, SD = 0.63; residents M = 3.39, SD = 0.66; *p* < 0.0001). As can be seen, clinical teachers rated their busyness higher than the residents did, and the residents reported caring more about the personal characteristics of the teacher, such as the teacher’s personality, relevant medical knowledge, and ability to give feedback, as well as their relationship with them.

## Discussion

We surveyed 144 clinical teachers and residents in multiple EDs, asking them to report on the context, frequency, satisfaction, and influential factors of feedback provision. We specifically examined some of the areas unique to clinical feedback rather than assessment feedback, such as the competing demands of maintaining clinical work and patient safety and the relationship between the seeker and the provider. If feedback is not an anticipated and mandatory teaching moment, as it is in a formative assessment, the above considerations can play an important role in determining whether, when, and how feedback is provided.

In our study, the resident group frequently reported seeking feedback when they were deciding on a patient’s disposition. Disposition in an emergency medicine setting refers to deciding where the patient goes after initial ED management, such as discharge, admission, or observation. Junior doctors often need suggestions from their clinical teachers to make these kinds of final decisions. Other situations in which feedback is sought include when encountering unusual or rare medical cases or when a patient’s condition changes, all indicating that seeking feedback is an important part of clinical learning in the context of uncertainty. This kind of feedback is learner-initiated and driven by learning needs, which differs from the usual post-event feedback that commonly occurs in teacher-provided debriefings [32]. Furthermore, although it was requested, real-time feedback was not incorporated as part of usual practice in our study. This is not uncommon. For example, Piqette et al. [33] analyzed observations of the interactions between trainees and their supervisors that represented *overt learning opportunities* for the trainees across 74 acute care episodes within critical care wards. They found that, when dealing with clinical uncertainty during complex and changing medical situations, the supervisors found it hard to initiate overt teaching interactions, and when they did, interruptions occurred (e.g., patients’ conditions changed rapidly) preventing any meaningful feedback for trainees.

The clinical teachers in our study were not very satisfied with the feedback they provided. Given that ED is one of the most crowded clinical settings for medical education, it is not surprising to find that *being busy* was listed as the top influential factor by the clinical teachers. In examining the different weightings given by the clinical teachers and the residents on the *being busy* item, it became clear that it was the attending physicians, who hold the legal responsibility for patients, who were more stressed under the pressure of their clinical workloads. In addition, the clinical teachers commonly reported providing feedback when they felt a resident’s decision might be harmful to a patient and when a patient’s condition changed. Indeed, the duration and frequency of the average feedback in our study (1–5 min, 1–5 times per shift) corresponded with the impression that feedback provision in a busy clinical setting is often circumstantial, unpredictable, short, and to the point [26]. These results appeared to reveal the inner concerns of the busy clinical teachers about patient safety—and the constraint of only being able to deliver essential feedback in the moment and no more. Tailored faculty development courses aimed at providing training on giving efficient feedback in action [34] should be developed for educators who need to cope in busy clinical settings.

Another important finding was that both groups reported that personality, clinical knowledge, feedback ability, and learning attitude were important concerns when considering the seeking or provision of feedback. This is similar to previous research findings and highlights the interactional and reciprocal nature of clinical feedback provision [19, 35]. Telio et al. [25] proposed the important concept of the “educational alliance” in their article, suggesting that both context and relationships in the feedback process are important. They further indicated that it is also important for both parties to have mutual trust and an understanding of the learner’s role and goals. While many clinical medical education programs are not commonly designed in a way that supports an education alliance model [11], the results of our—and other—studies suggest that cultivating an educational alliance between faculty and learners and building close relationships based on good intentions are as important as the facets of faculty development and efforts to improve coaching and mentoring techniques [23].

In terms of the participants’ perceptions, we found that the clinical teachers in our study were more likely to report that they initiated the feedback process than the learners did, and being *too busy* was the highest-rated barrier to the provision of clinical feedback. This resonates with the findings of Yarris et al. [15] who reported on their large-scale questionnaire study conducted in the US. However, there are some differences. For example, in Yarris et al.’s study, a significantly higher proportion of clinical teachers rated their feedback quality as being *very good* or *excellent*, whereas in our study, the clinical teachers rated their satisfaction as being significantly lower than that of the residents in every aspect of their clinical feedback. One possible explanation for this could be that the learners in our study overrated the feedback they received. Indeed, research suggests that, in a high power distance culture (i.e., one in which the differentials of power are accepted rather than contested), such as in Confucian Asian countries, students are often more reflective, satisfied with the lessons offered to them, and lack motivation (or confidence) to challenge the existing educational system [36, 37]. Alternatively, it could be that the clinical teachers in our study underrated their feedback, possibly due to a lack of confidence or modesty.

Researchers have previously suggested a number of *hows* for feedback provision in busy settings, including that it should be based on first-hand data, focused on specific performance, well-timed, and delivered in measured amounts [18, 38]. Educating clinicians to recognize the many opportunities to give feedback that are presented to them as a teaching tool has also been proposed [7], and evidence suggests that feedback quality can be improved by educational interventions, including simulation [39, 40]. However, our recommendations for practice focus on both the *when* and *how*; thus, we suggest that educators also consider situations of uncertainty for their residents, such as when deciding on the patient’s next step or when communication problems are likely to occur. By providing feedback in anticipation of those situations or in real time, valuable learning opportunities will open up for the betterment of both residents and patients.

### Limitations

As with any research, our study had a number of limitations that should be considered when attempting to generalize across settings. This was a single-nation, multicenter study. As the medical systems and clinical teaching and learning environments vary between countries, the results of the current study may not be generalizable to another ED in a different country. Indeed, the survey was conducted in an Asian culture and comprised self-reported items: some participants may therefore have given answers that would be desired instead of reporting what actually happened or how they actually felt. Further on-site or videotape observational study may be needed to address this self-report issue. Lastly, although the response rate was relatively high (80%), the voluntary online questionnaire format may have been subject to selection bias because the physicians who care about the issue of clinical feedback may have been more motivated to participate in the study.

## Conclusions

The provision of clinical feedback in the ED is often short, circumstantial, and mainly initiated by clinical teachers. Furthermore, positive feedback is generally less expected. Providing or seeking feedback appears to be an important part of clinical learning in the context of uncertainty. In our study, various suitable situations for feedback provision were identified, and the effects of influential factors on learners and clinical teachers were quantified and compared. The importance of the relationship between the feedback seeker and provider highlights the interactive and reciprocal nature of clinical feedback provision. The results of this study could be incorporated into faculty development courses to enhance clinical feedback provision in busy clinical environments.

## Additional file

Additional file 1: English translation of Questionnaire. (DOCX 44 kb)
