# Supplementary material for: Clinical learning in the context of uncertainty: a multi-center survey of emergency department residents’ and attending physicians’ perceptions of clinical feedback
Source: BMC Med Educ. 2019 May 29;19:174. doi: 10.1186/s12909-019-1597-8 (PMC6542138; doi:10.1186/s12909-019-1597-8)
Supplement: Supplementary file 1 — English translation of Questionnaire. (DOCX 44 kb) [file 12909_2019_1597_MOESM1_ESM.docx]

1. Supplementary material

**Translation of Questionnaire**

| **Clinical teacher Version** (basic information excluded) |
| --- |
| **Please answer the following questions according to your usual experience of feedback.** |
| Who usually initiates the giving of feedback?  □ Me (proactively gives feedback)  □ Resident (proactively asks for feedback)  □ Fifty fifty (Both the resident and I) |
| The duration in which I gave feedback to the resident usually lasted for an average of …  □ <1 minute □ 1~5 minutes □ 6~10 minutes □ >10 minutes |
| Under what circumstance would you usually give feedback to the resident ? (you can choose multiple answers)  □ When encountering unusual or rare medical cases  □ When the resident’s decision (order) can cause harm to the patient  □ When the resident takes the initiative to seek feedback  □ After a resident was observed to perform well  □ When there are communication problems  □ When the resident had done his/her initial evaluation / assessment of the patient  □ After medical orders were given by the resident  □ When there are new findings to the patient’s examination results  □ When there are changes to the patient’s condition  □ When deciding on the patient’s disposition  □ Others. Please specify |
| How often do you give feedback to the resident ?  □ ≥5 times during each shift  □ <5 times during each shift  □ I do not give feedback during each shift – about 1 feedback every 2-3 shifts  □ I do not give feedback during each shift – only 1 feedback after more than 3 shifts |
| **From your experience in giving feedback to resident s, please select the option that best describes the quality of the feedback that you gave for each feedback component. (Very good, Good, Poor, Very poor)** |
| Positive feedback (when the resident performed well) |
| Negative feedback (to correct the mistakes of the resident) |
| Imparting medical knowledge |
| Improving communication skills |
| Development of professionalism |
| Improving clinical skills |
| Medical charting |
| Patient care |
| Making evidence-based medical decisions |
| **Overall level of satisfaction (Strongly agree, agree, disagree, strongly disagree)** |
| Overall, I am satisfied with the “quality of feedback” that I am giving. |
| Overall, I am satisfied with the “number of times” that I am giving feedback. |
| Overall, I am satisfied with the “timeliness” at which I am giving feedback. |
| **From your experience in giving feedback to resident s, please select the option that best describes how each factor below influences your willingness to give feedback. (Strongly agree, Agree, Disagree, Strongly disagree)** |
| The learning attitude of the resident |
| The personality of the resident |
| The clinical skills of the resident |
| My familiarity with the resident |
| My medical knowledge regarding specific problems |
| My ability to give feedback |
| Past experience of having given feedback which did not go well |
| Being busy |
| Worry that wrongly correcting the mistake of the resident will affect our relationship |
| Sudden events at the emergency room which disrupts the giving of feedback |
| The emergency room’s environment which is open and lacks privacy |
| **From your experience in giving feedback to residents, please select the option that best describes whether you would change the way you give feedback based on each factor below. (Strongly agree, Agree, Disagree, Strongly disagree)** |
| The level of the resident |
| The learning attitude of the resident |
| The personality of the resident |
| The clinical skills of the resident |
| My familiarity with the resident |
| Past experience of having given feedback which did not go well |
| **From your experience in giving feedback to resident s, please answer the questions below. (Strongly agree, Agree, Disagree, Strongly disagree)** |
| I will forget to give the resident feedback when I am too focused at work |
| The need to handle the stress when working in the emergency room (non-teaching class) |
| I will hesitate between “giving feedback” and “wanting to finish my clinical work” |
| Having to adapt to different attending physicians everyday is troublesome. |
| I do not want to get off work late because I have to give feedback. |

| **Residents Version** (basic information excluded) |
| --- |
| **Please answer the following questions according to your usual experience of feedback.** |
| Who usually initiates the giving of feedback?  □ Attending (proactively gives feedback)  □ Me (proactively asks for feedback)  □ (Both the Attending and I) |
| The duration in which I received feedback usually lasted for an average of … / How long did the duration in which you received feedback lasted on average?  □ <1 minute □ 1~5 minutes □ 6~10 minutes □ >10 minutes |
| Under what circumstance would the Attending usually give feedback to you? (you can choose multiple answers)  □ When encountering unusual or rare medical cases  □ When my decision can cause harm to the patient  □ When I take the initiative to seek feedback  □ When I think that I have handled the patient well  □ When there are communication problems  □ After I done my initial evaluation of the patient  □ When I had written the order / After medical orders were given by Me  □ When there are new findings to the patient’s examination results  □ When there are changes to the patient’s condition  □ When deciding on the patient’s disposition  □ Others. Please specify |
| How often do you receive feedback?  □ ≥5 times during each shift  □ <5 times during each shift  □ I do not receive feedback during each shift – about 1 feedback every 2-3 shifts |
| **From your experience in receiving feedback from Attendings, please select the option that best describes the quality of the feedback that was given to you for each feedback component.** |
| Positive feedback (when I performed well) |
| Negative feedback (to correct my mistakes) |
| Imparting medical knowledge |
| Improving communication skills |
| Development of professionalism |
| Improving clinical skills |
| Writing medical records |
| Patient care |
| Making evidence-based medical decisions |
| **Overall level of satisfaction (Strongly agree, Agree, Disagree, Strongly disagree)** |
| Overall, I am satisfied with the “quality of feedback” that I am receiving. |
| Overall, I am satisfied with the “number of times” that I am receiving feedback. |
| Overall, I am satisfied with the “timeliness” at which I am receiving feedback. |
| **From your experience in receiving feedback from Attendings, please select the option that best describes how each factor below influences your willingness to seek feedback. (Strongly agree, Agree, Disagree, Strongly disagree)** |
| The personality / attitude of the Attending |
| The medical knowledge of the Attending |
| The ability of the Attending to give feedback |
| My familiarity with the Attending |
| Past experience of having received feedback which did not go well |
| Being busy |
| Sudden events at the emergency room which disrupts the receiving of feedback |
| The emergency room’s environment which is open and lacks privacy |
| The different types of shift (i.e. teaching or practical class) |
| **From your experience in receiving feedback from Attendings, please answer the questions below. (Strongly agree, Agree, Disagree, Strongly disagree)** |
| Having to adapt to different attending physician everyday is troublesome. |
| I will hesitate between “seeking feedback” and “wanting to finish my clinical work” |
| I will change the way I work according to the style of each Attending. |
| I do not want to get off work late because I have to seek feedback. |
